# An Immunocompetent Microphysiological System to Simultaneously Investigate Effects of Anti-Tumor Natural Killer Cells on Tumor and Cardiac Microtissues

**DOI:** 10.3389/fimmu.2021.781337

**Published:** 2021-12-02

**Authors:** Oanh T. P. Nguyen, Patrick M. Misun, Christian Lohasz, Jihyun Lee, Weijia Wang, Timm Schroeder, Andreas Hierlemann

**Affiliations:** ^1^ Bio Engineering Laboratory, Department of Biosystems Science and Engineering, ETH Zürich, Basel, Switzerland; ^2^ Cell Systems Dynamics Group, Department of Biosystems Science and Engineering, ETH Zürich, Basel, Switzerland

**Keywords:** microphysiological system, 3D microtissue, natural killer cell, adoptive cell therapy, efficacy and safety assessment

## Abstract

Existing first-line cancer therapies often fail to cope with the heterogeneity and complexity of cancers, so that new therapeutic approaches are urgently needed. Among novel alternative therapies, adoptive cell therapy (ACT) has emerged as a promising cancer treatment in recent years. The limited clinical applications of ACT, despite its advantages over standard-of-care therapies, can be attributed to (i) time-consuming and cost-intensive procedures to screen for potent anti-tumor immune cells and the corresponding targets, (ii) difficulties to translate *in-vitro* and animal-derived *in-vivo* efficacies to clinical efficacy in humans, and (iii) the lack of systemic methods for the safety assessment of ACT. Suitable experimental models and testing platforms have the potential to accelerate the development of ACT. Immunocompetent microphysiological systems (iMPS) are microfluidic platforms that enable complex interactions of advanced tissue models with different immune cell types, bridging the gap between *in-vitro* and *in-vivo* studies. Here, we present a proof-of-concept iMPS that supports a triple culture of three-dimensional (3D) colorectal tumor microtissues, 3D cardiac microtissues, and human-derived natural killer (NK) cells in the same microfluidic network. Different aspects of tumor-NK cell interactions were characterized using this iMPS including: (i) direct interaction and NK cell-mediated tumor killing, (ii) the development of an inflammatory milieu through enrichment of soluble pro-inflammatory chemokines and cytokines, and (iii) secondary effects on healthy cardiac microtissues. We found a specific NK cell-mediated tumor-killing activity and elevated levels of tumor- and NK cell-derived chemokines and cytokines, indicating crosstalk and development of an inflammatory milieu. While viability and morphological integrity of cardiac microtissues remained mostly unaffected, we were able to detect alterations in their beating behavior, which shows the potential of iMPS for both, efficacy and early safety testing of new candidate ACTs.

## Introduction

The lack of treatment options renders cancer one of the major health burdens of our time. The International Agency for Research on Cancer ranks cancer the second leading cause of death, with an estimated global impact of 19.3 million new cancer cases and approximately 10 million cancer deaths in 2020 alone (1). Current standard cancer treatments, i.e., radio- and chemotherapies as well as surgery are still confronted with multiple setbacks. While non-invasive approaches suffer from severe side effects, low efficacy, and therapy resistance, invasive surgery is only applicable for a limited number of localized and contained solid tumors (2). The search for safer and more durable therapies led to the interdisciplinary efforts in the fields of oncology and immunology and the development of cancer immunotherapies. Since the first description of immunotherapy in the 1980s (3), a large number of immunotherapeutic approaches have recently entered clinical evaluation (4). These novel therapies utilize different components of the immune system, such as antibodies or immune cells to instruct the patient’s immune system to target the cancer cells (5, 6). Among emerging cancer immunotherapies, adoptive cell therapy (ACT) – a cell-based immunotherapy – holds promise to personalize immunotherapy for each patient’s condition. Cytotoxic immune cells, such as CD8^+^ T cells or natural killer (NK) cells are isolated from patients (autologous) or healthy donors (allogeneic). The cells are expanded *in vitro* and, in some cases, genetically engineered to increase their lifespan and *in-vivo* tumor-killing activity. High numbers of these immune cells are then transferred back into the patient to mediate anti-tumor activity (7). Although ACT offers an alternative treatment option for cancer patients, who are refractory to standard therapies, clinical trials of ACT with satisfactory results have been limited to hematologic malignancies (7, 8). For non-hematologic solid tumors, positive outcomes of such therapies are sporadic. For instance, despite of its success to suppress leukemia (9), NK cell-based ACT did not show any activity against metastatic melanoma in a clinical trial by Parkhurst et al. (10). It is worth mentioning that this clinical trial for ACT, and many other trials, were carried out after substantial *in-vitro* testing. The high anti-tumor activity evidenced in pre-clinical *in-vitro* screenings and the contrasting lack of efficacy afterwards *in vivo* highlight the poor *in vitro-to-in vivo* translatability of complex treatments. Such poor translatability has been attributed mainly to the widespread use of conventional two-dimensional (2D) cell cultures and animal models for pre-clinical evaluations (11).

Traditional 2D cell cultures fail to mimic the architecture and cellular heterogeneity of a solid tumor and cannot realistically recapitulate tumor-immune cell interactions. Likewise, animal models fail to reliably predict the efficacy and safety of immune-cell-based therapies due to critical immunological differences between animals and human beings (12, 13). During the past two decades, human cell-derived 3D tissue models have attracted more attention as tumor models for therapy screening as they overcome problems associated with 2D cell cultures. Under carefully designed culture conditions, tumor cells can form 3D microtissues (MTs) that are spherical, compact, and closely resemble *in-vivo* tumors in terms of structure, metabolism, loss of polarized cell morphology – as found in epithelial tissue-originated tumors, and gene-expression profiles (14).

Microphysiological systems (MPSs) combine advanced tissue models, such as 3D MTs, organoids or bioartificial tissues with microfluidic technology. Such systems are key innovations to further develop and refine advanced tissue models. The microfluidic components within MPSs can be designed to mimic different aspects of a tissue’s microenvironment, such as physical and mechanical cues, and allow for interconnection of several tissue models (15). Currently developed MPSs can interconnect up to ten organ models for an experimental duration of up to four weeks (16, 17), making them suitable systems for systemic investigations of inter-tissue communication and for therapeutic testing. A wide range of single- or multi-tissue MPSs have been developed, among which are lung MPSs (18), gastrointestinal MPSs (19), liver MPSs (20), and immunocompetent MPSs (iMPSs).

The majority of reported iMPSs for immune-oncology purposes included either single tumor cells or 3D tumor MTs (TuMTs) that were embedded in hydrogel. Immune cells were added into microfluidic channels adjacent to the hydrogel, which were initially separated from the tumor cells (21). Such a configuration mimics the placement of cell components in the tumor microenvironment (TME). The hydrogel recapitulated the dense interstitial extracellular matrix (ECM) mesh of an *in-vivo* TME that immune cells have to penetrate to reach the tumor cells. Such realistic configurations helps to avoid overestimations of anti-tumor efficacy – which are likely to be obtained with systems that combine immune and tumor cells and enforce mutual interaction (22, 23). Furthermore, 3D constructs and iMPSs can help to mimic processes, such as immune-cell recruitment and migration, tumor infiltration, and TME-relevant immunosuppression (22, 24–26) that cannot be studied with conventional 2D cell cultures. Although it could be shown that TME can influence therapeutic outcomes, the indispensable use of ECM hydrogel limits the experimental readout options to microscopy measurements. Additionally, most studies focused on demonstrating treatment efficacy while the safety assessment of candidate ACTs was neglected. Two major risk factors of ACT include (i) on-target, off-tumor attack of healthy cells by cytotoxic immune cells, and (ii) the high level of soluble inflammatory chemokines and cytokines that are released during tumor recognition and elimination. Cytotoxic immune cells recognize tumor cells *via* pairing between specific sets of their surface receptors and corresponding ligands on the tumor cell surface. However, most of these ligands are also expressed on healthy cells, which can result in accidental on-target, off-tumor attack by these immune cells (27). Moreover, tumor-immune cells interactions can give rise to a complex of inflammatory chemokine and cytokines, eventually creating an inflammatory environment that is harmful to bystander organs (27–29). These adverse effects are difficult to predict even with animal models (30). Currently, most ECM hydrogel-based iMPSs are also not capable to simultaneously assess drug efficacy on the tumor and its toxicity on secondary, healthy organs.

**Figure 1:**
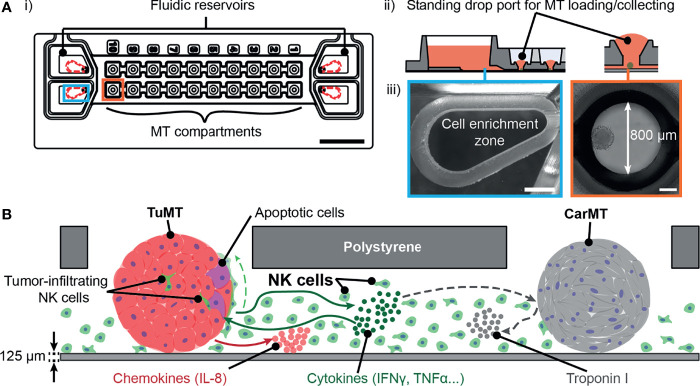
**(A)** i) A schematic drawing of the iMPS, which is based on the Akura™ Flow platform (modifications indicated as red dashed lines). Scale bar: 10 mm. ii) Cross-sectional view of one reservoir and adjacent MT compartments. iii) Bright-field images of the cell enrichment zone inside one reservoir (scale bar: 1 mm) and a MT compartment with a TuMT (scale bar: 200 μm). **(B)** Schematic representation of on-chip cell cultures and possible interactions among components.

In an effort to narrow the gap between *in-vitro* studies and the *in-vivo* situation, we developed an iMPS, which allows for co-culturing of anti-tumor immune cells and 3D MTs. With this system, we aim at addressing current limitations of iMPS, such as the local confinement of immune cells in hydrogels, the low experimental throughput due to technical complexity, and missing models of healthy tissues for simultaneous toxicity testing. We used umbilical cord blood (UCB)-derived NK cells, whose anti-tumor activity involves both, direct interaction of NK cells with tumor cells and indirect tumor suppression *via* chemokine/cytokine signaling (31, 32). The 3D tumor model was established from the colorectal tumor cell line HCT116, the cells of which can produce their own ECM (33) and form compact, solid tumor-like MTs (TuMTs). 3D cardiac MTs (CarMTs) – formed from induced pluripotent stem cell (iPSC)-derived cardiac myocytes – were chosen as healthy-tissue model. All organ models were combined in the microfluidic chip that was developed for culturing of suspension cells and several, spatially separated, solid tissue models. Dedicated cell enrichment zones confined NK cells inside the medium reservoirs at the ends of the microfluidic channels (**Figure 1A**). During the experiments, NK cells either stayed in the cell enrichment zones or circulated back and forth along the same microfluidic channel (**Figure 1A**, ii and iii). Medium perfusion was actuated by gravity-driven flow by tilting the microfluidic chips, which ensured a constant exchange of soluble factors between the three tissue types. Different indicators of tumor-NK cell interaction were used: (i) NK cell-induced apoptosis of tumor cells, (ii) an elevated level of inflammatory chemokines [interleukin-8 (IL-8)] and cytokines [interferon-γ (IFN-γ), tumor necrosis factor-α (TNF-α), granulocyte-macrophage colony-stimulating factor (GM-CSF)], produced by TuMTs and NK cells, and (iii) invasion of NK cells into the TuMT volume (**Figure 1B**). To study the health status and detect structural damages of CarMTs, we recorded and analyzed the pattern of their spontaneous beating and measured soluble Troponin I in the cell culture supernatant. Our iMPS can potentially be used for early recognition of ACT-associated cardiotoxicity, particularly for NK cell-based ACT, the causes and consequences of which are still under investigation (34, 35).

## Materials and Methods

### Microfluidic Chip

We modified the Akura™ Flow MPS discovery platform (InSphero, Schlieren, Switzerland), which was originally developed to study inter-tissue communication between 3D MTs (36). The microfluidic chip features two individual microfluidic channels with medium reservoirs at both ends. Each channel can accommodate up to ten fluidically interconnected MTs, which are located in the MT compartments (**Figure 1A**, i). To accommodate NK cells in suspension and to promote their direct interaction with 3D MTs, we adapted the chip by computer numerical control (CNC) micro-milling: (i) We introduced a drop-shaped cell-enrichment zone in the medium reservoirs (**Figure 1A**, ii and iii, left panels). The cell enrichment zone retained NK cells close to the entrance to the microfluidic channel after each tilting cycle and prevented them from accumulating in the low-flow zones in the corners of the reservoirs. For gravity-driven flow-based experiments, each microfluidic channel was supplied with 200 µL of fresh medium every day. This enabled the use of enough cell-culture medium to maintain all tissue models viable during the culturing periods. (ii) To facilitate direct cell-cell interactions between NK cells and MTs, we removed the barrier structures in the MT compartments (**Figure 1A**, ii and iii, right panels) and enlarged the microfluidic channels to a cross-section of 220 μm × 600 μm (height × width). More details on the performed modifications are shown in **Figure S1**. Gravity-driven perfusion was induced by tilting the chip back and forth over a tilting angle of ±5° using the Akura™ Flow system (InSphero) inside a standard cell-culture incubator. Each tilting cycle included a 5-min halt at the positions of maximum tilting angle in both directions and a 1 h 40 min halt in a horizontal position. Detailed protocols for MT loading and system operation were also previously described by Lohasz et al. (36) and are demonstrated in **Video S1**.

### Cell Cultures

#### Formation of 3D Tumor and Cardiac MTs

All cell cultures were maintained in a humidified incubator at 37°C and 5% CO_2_ (Binder CB 220, Tuttlingen, Binder, Germany). The HCT116 human colorectal carcinoma cell line (ATCC^®^ CCL-247) was purchased from the American Type Culture Collection (ATCC, Manassas, VA, USA). In brief, cells were cultured in cell culture flasks using a tumor-growth medium that contains Roswell Park Memorial Institute (RPMI) 1640 medium (BioConcept, Allschwil, Switzerland), 10% heat-inactivated fetal bovine serum (h.i. FBS; Gibco, Thermo Fisher Scientific, Waltham, MA, USA), 2 mM CTS™ GlutaMAX™ supplement (Gibco, Thermo Fisher Scientific), 1 mM sodium pyruvate (Gibco, Thermo Fisher Scientific), 1× non-essential amino acids (NEAA) (Merck, Darmstadt, Germany), and 50 μg/mL Kanamycin (BioConcept). Medium exchange was done every two days, and the cells were sub-cultured when reaching approximately 85% confluence.

The hiPSC line, CW30318CC1 (healthy donor, female), was obtained from the CIRM hPSC Repository funded by the California Institute of Regenerative Medicine (CIRM) *via* FujiFilm Cellular Dynamics (Madison, WI, USA). This cell line was differentiated to cardiac myocytes using the PSC Cardiomyocyte Differentiation Kit (Gibco, Thermo Fisher Scientific). iPCS-derived cardiac myocytes were maintained as monolayers in standard 12-well plates (Greiner Bio-One, Kremsmünster, Austria), pre-coated with Geltrex extracellular matrix (Gibco, Thermo Fisher Scientific) – diluted 1:50 in PBS without Ca^2+^ and Mg^2+^ (Gibco, Thermo Fisher Scientific). Medium exchange was performed twice a week with a cardiac myocyte growth medium that contains RPMI 1640, 2 mM CTS™ GlutaMAX™ supplement, 1× B27 supplement (Gibco, Thermo Fisher Scientific), and 50 μg/mL Kanamycin. No passaging was performed during cardiac myocytes maintenance as the cardiac myocytes hardly divide in culture. Only prior to MT formation, cells were lifted with TrypLE Express enzyme solution (Gibco, Thermo Fisher Scientific) for cell suspension preparation. Here, TrypLE Express enzyme solution was used to preserve the expression of cell surface markers (37).

For 3D MT off-chip production and maintenance, Nunclon™ Sphera™ U-shaped-bottom, 96-well plates (96U-well plates) (Thermo Fisher Scientific) were used. 3D TuMTs were formed from the HCT116 cell line in tumor-growth medium at an initial seeding density of 500 cells/MT. In brief, 100 μL of cell suspension containing 5000 cells/mL were seeded to each well of a 96U-well plate and spun down at 250 ×g for 2 min. TuMTs were ready to use at day 4 post seeding when their diameters reached approximately 400 μm. At this size, the necrotic core did not form yet, and the TuMTs were large enough to not escape the MT compartments.

We formed CarMTs in the cardiac myocyte growth medium using an initial seeding density of 6500 cells/MT. Cardiac myocyte suspension was prepared in cardiac myocyte growth medium, supplemented with 20% h.i. FBS. Then, 200 μL of the prepared suspension were seeded to each well of a 96U-well plate and spun down at 200 ×g for 3 min. After 24 h, a compact cell cluster formed, and the medium was replaced with standard cardiac myocyte growth medium. Spontaneous beating of CarMTs typically started between day 3 and day 4. To ensure reproducibility among experiments, we only used CarMTs from day 5 post seeding, when beating activity was observed in 100% of MTs. Regular microscopy inspection was carried out, and CarMTs with a weak beating activity or abnormal shapes were disqualified. CarMT size attained roughly 380 μm at day 5 post seeding with a slight shrinkage (~10-20 μm in diameter) over time due to compaction. Once formed, CarMTs can be maintained up to one month with medium exchange twice a week. During all preparation steps, all cells were kept at 37°C on a thermostat plate. Both types of MT were imaged with a Cell3iMager Neo plate scanning system (SCREEN Group, Kyoto, Japan) for quality check before each experiment.

#### NK Cells

##### Ethical Statement

Anonymized human umbilical cord blood (UCB) samples were collected from healthy newborns of both sexes at the University Hospitals Basel with parental informed consent. Relevant ethical regulations were followed, according to the guidelines of the local Basel ethics committee (vote 13/2007V, S-112/2010, EKNZ2015/335).

##### Sample Processing and Cell Isolation

After collection, UCB cells were processed by density gradient centrifugation. CD34 positive (CD34^+^) and negative (CD34^-^) cells were separated using EasySep CD34 positive selection kit II (StemCell Technologies, Vancouver, BC, Canada) and cryopreserved.

NK cells were isolated from the cryopreserved CD34^–^fraction (hematopoietic stem cells removed) of human umbilical cord blood (UCB). We used the EasySep NK cells isolation kit (StemCell Technologies) to isolate NK cells and maintained them in NK cell-growth medium (RPMI 1640, supplemented with 10% h.i. FBS, 2 mM CTS™ GlutaMAX™ supplement, 1 mM sodium pyruvate, 1× non-essential amino acids (NEAA), 50 μg/mL Kanamycin, 50 μM β-mercaptoethanol (Gibco, Thermo Fisher Scientific), and 200 U/mL recombinant human interleukin-2 (IL-2; Peprotech, Cranbury, NJ, USA)) for up to two weeks. Fluorescein isothiocyanate (FITC)-conjugated CD45 (clone HI30), Phycoerythrin (PE)-conjugated CD3 (clone UCHT1), and Allophycocyanin (APC)-conjugated CD56 antibodies (clone HCD56) – all were purchased from StemCell Technologies – were used to confirm the purity of NK cells after isolation by flow cytometry (BD Fortessa, BD Biosciences, Franklin Lakes, NJ, USA). Additionally, 2-(4-amidinophenyl)-6-indolecarbamidine dihydrochloride (DAPI) stain (Merck) was used to assess cell viability in flow cytometry analysis. Where indicated, NK cells were transferred to an NK cell-activating medium that contained 1000 U/mL IL-2 and 20 ng/mL of recombinant human interleukin-15 (IL-15; Peprotech) for 5 days before the experiments with a partial medium exchange at day 3. This pre-treatment was extensively used to enhance the overall proliferation and cytotoxic activity of NK cells against the target tumor (38, 39), especially before on-chip cultures.

### Cell Labeling and Live-Cell Imaging

To spatially track NK cells within the chip, we labeled the cells with Cytopainter Cell Proliferation Staining Reagent – Green fluorescence, (Abcam, Cambridge, UK), diluted from 500× stock solution in NK cell-growth medium, for 40 min at 37°C before seeding them into the iMPS. BioTracker NucView Blue 405 Caspase-3 Dye (PBS) (Merck) was added directly into the cell-culture medium with a final concentration of 5 μM to visualize apoptotic cells during the experimental duration. Live-cell imaging was performed on a fluorescence Nikon TiE microscope (Nikon Europe B.V., Amsterdam, Netherlands) every day with a Plan Fluor 10× objective.

### Static, Well Plate-Based Cultures of NK Cells and MTs

For static co-culture experiments, we combined NK cells with each type of MT in a 96U-well plate to assess the cytolytic activity and the cytokine release of isolated NK cells. Since HCT116 cells are relatively resistant to NK cell-induced cytolysis at a low effector-to-target (E:T) ratio (40), we used a high E:T ratio of 10:1 based on the initial seeding density of HCT116. First, the culture wells were pre-loaded with 100 μL of NK cell-growth medium, into which pre-formed MTs were transferred by contact transfer. Then, the wells were topped with 100 μL of NK cell suspension prepared in the same medium. For mono-cultures, the wells were filled with equal volumes of NK cell-growth medium without cells. The plate was placed inside a cell-culture incubator for three days without medium exchange. The morphological changes of MTs were monitored daily by bright-field imaging. MTs and the cell-culture supernatant were collected every day for performing viability assays and chemokine/cytokine quantitations. Jurkat cells, clone E6-1 (ATCC) were co-cultured with MTs using the same experimental layout as a negative control for tumor-killing activity. As positive controls for cardiotoxicity, CarMTs were treated with 30 μM Doxorubicin hydrochloride (Dox; Tocris, Bristol, UK) for 3 days in a Nunclon Sphera 96U-well plate before measuring Troponin I levels (41).

### On-Chip Cultures in iMPS

TuMTs and CarMTs were transferred to the iMPS chip by using a contact-transfer technique at day 4 and at day 5 post seeding. Each microfluidic channel was loaded with six TuMTs and four CarMTs. Phenol red-free NK cell-growth medium was used for all on-chip cultures. Fluorescently labeled NK cells were spun down at 500 ×g for 5 min at 4°C and resuspended in a pre-warmed medium at a density of 1.67 × 10^6^ cells/mL. Since a local administration of NK cells has proven to increase the amount of NK cells at the tumor site and can lead to better tumor suppression (42), we introduced the NK cell suspension directly into MT compartments through their loading ports. A total amount of 30 μL of NK cell suspension was loaded in 5 μL-dispensing steps into each TuMT-containing MT compartment. The chip was kept in a horizontal position (without perfusion) for 3 hours to prime the interaction between NK cells and MTs. Fluorescence imaging was conducted at the end of the priming period to check the presence of NK cells inside the MT compartments and cell enrichment zones. On-chip cultures were maintained for 3 days in the Akura™ Flow system inside a cell culture incubator. To assess the beating activity of CarMTs, we recorded 20 second-long AVI videos of each CarMT with a frame rate of 100 frames per second at the beginning and at the end of the experiments. Medium was exchanged daily during 3 days, and the removed medium was stored at -20°C for supernatant-based assays. After the co-culturing period, all unbound NK cells were removed from the microfluidic chip, and MTs were either (i) collected from the chip for ATP-dependent viability assays using the CellTiter-Glo 3D cell viability assay (Promega, Madison, WI, USA) or (ii) fixed for high-resolution microscopy.

### Immunofluorescence (IF) Staining and High-Resolution Microscopy

MTs were fixed directly on chip after the experiment. In brief, all supernatant was removed from the reservoirs, then all microfluidic channels were flushed twice with 200 μL of phosphate-buffered saline (PBS, with calcium chloride (Ca^2+^) and magnesium chloride (Mg^2+^), Merck). Then, 100 μL of 2% formaldehyde in PBS (Merck) were added to the microfluidic channels for 10 min. All channels were flushed again three times with 200 μL of PBS (without Ca^2+^ and Mg^2+^; Gibco), and MTs were blocked with 5% bovine serum albumin (BSA; Merck) in PBS (without Ca^2+^ and Mg^2+^) for at least 1 hour. Depending on the experiments, different combinations of the following antibodies were used: Alexa Fluor (AF) 647-conjugated anti-Cytokeratin 18 (CK18; clone C-04; Santa Cruz Biotechnology, Dallas, TX, USA) – 1:50 dilution, AF594-conjugated polyclonal anti-CD69 (Bioss Antibodies, Woburn, MA, USA) – 1:200 dilution, and AF647-conjugated anti-human major histocompatibility complex (MHC) class I chain-related protein A and B (MICA/B) (clone 6D4, BioLegend, San Diego, CA, USA) – 1:50 dilution. All antibodies were diluted in 0.1% BSA in PBS (without Ca^2+^ and Mg^2+^) and incubated with the MTs overnight at 4°C. The washing step was repeated and, when applicable, nuclear counterstaining was performed using NucBlue™ Live ReadyProbes™ Reagent (Hoechst 33342, Invitrogen, Thermo Fisher Scientific). We used a non-hardening mounting medium [ibidi Mounting Medium (Ibidi, Gräfelfing, Germany)] to fill the whole system before imaging.

We acquired 190 – 200 μm-thick Z-stacks of MTs in 2-μm steps in different culture conditions to detect tumor-infiltrating NK cells using either an inverted Leica SP8 (Leica Microsystem, Wetzlar, Germany) or an inverted Nikon A1 (Nikon Europe B.V.) confocal laser scanning microscope. To inspect the expression of MICA/B NK cell ligand on the surface of TuMTs and CarMTs, 100 μm-thick Z-stacks of MTs were acquired in 0.4-μm steps using an X-Light v3 inverted spinning disk confocal microscope (Nikon Europe B.V.).

### Enzyme-Linked Immunosorbent Assay (ELISA)

Cell-culture supernatant was collected into a low-binding Nunc™ 96-well polypropylene storage microplate (Thermo Fisher Scientific). We centrifuged the plate at 2000 ×g for 10 min to remove cell debris, then transferred all supernatant to a new storage plate of the same type and stored the supernatant at -20 °C until use. We employed a customized bead-based multiplex assay according to the manufacturer’s protocol (BioRad, Hercules, CA, USA) to measure IL-8, GM-SCF, IFN-γ, and TNF-α inside the supernatant. Soluble Troponin I and soluble MICA (sMICA) were measured separately using a human cardiac Troponin I ELISA kit (Abcam) and a MICA human ELISA kit (Invitrogen, Thermo Fisher Scientific), respectively, according to the manufacturers’ protocol and a Tecan Infinite M1000 Pro plate reader (Tecan, Männedorf, Switzerland).

### Data Analysis

Microscope images were processed and analyzed using the Nikon NIS-Elements Advanced Research (Nikon Europe B.V.) or ImageJ software. Beating patterns of CarMTs were analyzed using the Musclemotion macro (43) in ImageJ (National Institution of Health, Stapleton, NY, USA). We used the Bio-Plex Manager software (BioRad) and Microsoft Excel (Redmond, WA, USA) to analyze data obtained from the multiplex assay and the Troponin I ELISA. This data was statistically analyzed with one-way or two-way ANOVA depending on the data set and visualized using GraphPad Prism 7 software (GraphPad Software, San Diego, CA, USA). Data obtained from sMICA ELISA assay was processed and statistically analyzed with GraphPad Prism 7. All statistical results were represented as mean ± standard deviation (SD) with a significance of P < 0.05, unless indicated differently.

## Results

### Static, Well Plate-Based Cultures of NK Cells and MTs

Human NK cells are characterized by the absence of surface markers CD3 and the presence of CD56 (CD3^-^/CD56^+^). Therefore, after isolation, we quantitated the proportion of CD3^-^/CD56^+^ cells in the obtained population using flow cytometry. **Figure S2** shows that the purity of CD3^-^/CD56^+^ cells in our samples was up to 99.2%. The isolated NK cell population also appeared to express CD56 at different relative levels, which reflected the maturity and differentiation state of the NK cells. CD56^bright^ NK cells with high CD56 surface expression are immature and less cytotoxic as compared to fully differentiated CD56^dim^ NK cells with lower CD56 surface expression. These immature CD56^bright^ NK cells, however, can become as potent as their mature, differentiated counterpart through additional cytokine treatment (39), hence the use of NK cell-activating medium in our experiments.

**Figure 2:**
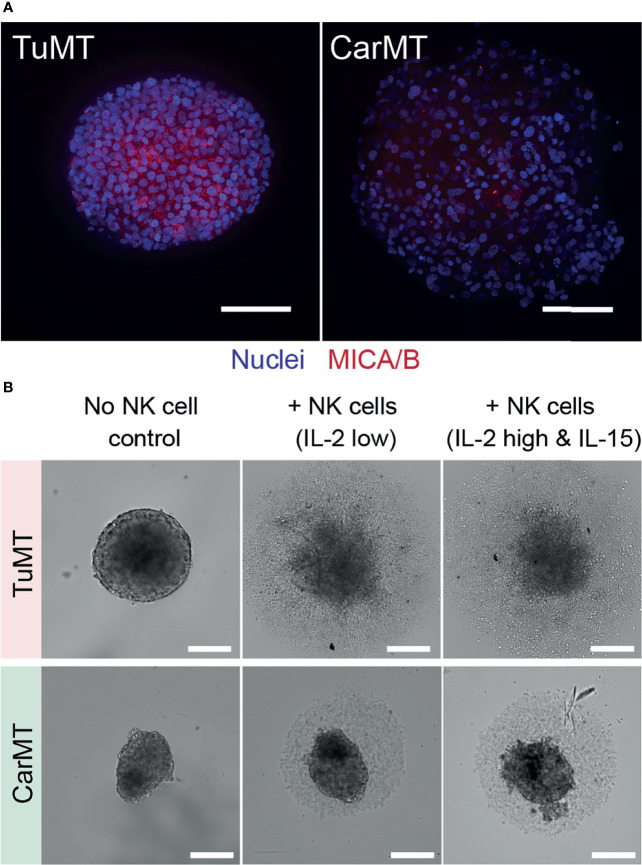
**(A)** Membrane-bound MICA/B expression on TuMTs and CarMTs shown by maximum intensity projection of 100 μm-thick Z-stacks. Scale bars: 100 μm. **(B)** Specific anti-tumor activity of UCB-derived NK cells in static co-cultures in a 96U-well plate. Upper panels: TuMT disintegrated after 72 hours in co-culture with NK cells. Lower panels: CarMT remained intact in co-cultures with UCB-derived NK cells. Scale bars: 200 μm.

The two selected solid tissue models, TuMTs and CarMTs, were qualitatively assessed for their ectopic expression of membrane-bound MICA/B. MICA/B are the most studied ligands for the NK group 2D (NKG2D) activating receptor, which is universally expressed by NK cells (44). **Figure 2A** shows high expression levels of membrane-bound MICA/B on tumor cells within the optically accessible outer layers of the TuMTs, while MICA/B was poorly expressed in CarMTs. These results are supported by other studies that report high expression levels of MICA/B on the cell surface of tumor cells but not on the surface of normal cells (45). Based on this result, we expected our UCB-derived NK cells to recognize and eradicate tumor cells, while CarMTs should remain mostly unaffected. In static co-cultures of each MT type and NK cells, we closely monitored the size change of the MTs and their chemokine/cytokine production to scrutinize the extent and specificity of NK-cell-mediated tumor-killing activity. Our results indicated that, in static TuMT-NK-cell co-cultures, UCB-derived NK cells showed specific anti-tumor activity against TuMTs, regardless of the cytokine treatments. As shown in **Figure 2B** (upper panel), TuMTs completely disintegrated after 3 days in TuMT-NK-cell co-cultures. The fast cytolysis of TuMTs occurred within the first day and was confirmed by the low intracellular ATP-dependent viability of the MTs and increased IFNγ concentrations, as compared to the low basal levels in mono-cultures of NK cells or TuMTs (**Figures S3A, B**). As expected, NK cells did not affect the morphology and viability of CarMTs after 3 days in co-culture as shown in **Figure 2B** (lower panel) and **Figure S3C**. IFN-γ levels in CarMT-NK-cell co-cultures were at least 10-fold lower than in co-cultures of NK cells and TuMTs (**Figure S3D**).

Negative control experiments with Jurkat cells, which do not have cytotoxic activity against TuMTs, showed that a certain additional mass of suspension cells did not interfere with the growth of TuMTs (e.g., through nutrient competition). No IFN-γ was detected in this co-culture (data not shown).

### iMPS: On-Chip Inter-Tissue Communication and Anti-Tumor Effects of NK Cells

To fully understand the dynamics and effects of each individual tissue model in our iMPS, we included multiple cell culture combinations, categorized into 3 groups as shown in **Table 1**: (i) mono-cultures of each individual tissue model, i.e., TuMTs, CarMTs, and NK cells, (ii) co-cultures of pairs of tissue models, and (iii) a triple culture that included all cell models. Data collected from mono-cultures were used as reference to assess the contributions of each tissue/cell type in the co-cultures and the triple culture, which revealed direct and/or indirect interactions.

**Table 1:** All cell culture conditions in the microfluidic on-chip cultures.

Culture condition	Abbreviation	Tissue model/combination
Mono-culture	Mono	1. TuMTs 2. CarMTs 3. NK cells
Co-culture	Co	1. TuMTs – NK cells 2. CarMTs – NK cells 3. TuMTs – CarMTs
Triple culture	Triple	1. TuMTs – CarMTs – NK cells

#### Tumor Growth

**Figure 3:**
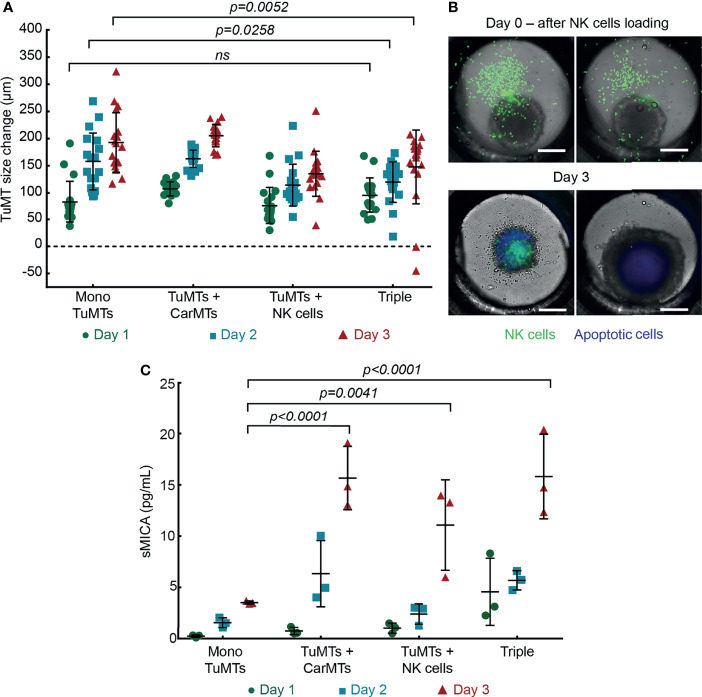
**(A)** TuMT size changes monitored by bright-field imaging. Diameters of individual TuMTs measured at Day 1 (D1), Day 2 (D2), and Day 3 (D3) were normalized to their own diameter at day 0 (n = 18 MTs) (Mono: mono-culture, Triple: triple culture). Detailed statistical comparisons between conditions are shown in **Figure S4A** (ns: not significant). **(B)** Representative fluorescence images reflecting heterogeneous size changes of TuMTs in triple cultures. NK cells were labeled with Green fluorescence cell proliferation staining reagent, while apoptotic cells were labeled with Blue 405 Caspase-3 Dye. Scale bars: 200 μm. **(C)** Quantitation of sMICA released into the supernatant of different cell cultures during a 3-day experiment (n = 3). Detailed statistical comparisons between conditions are shown in **Figure S4B**.

To obtain a first assessment on how CarMTs and/or NK cells affect TuMT growth in different culture conditions, we tracked the diameter of 18 individual TuMTs per cell culture condition every day during three days. Absolute TuMT size changes were calculated in reference to the size at day 0 of the experiment, at which the MTs were transferred to the chip. As shown in **Figure 3A** and **Table S1**, TuMTs grew steadily and similarly in mono-culture and in co-culture with CarMTs during the three days of the measurements.

In contrast, we observed heterogeneous changes in TuMTs size when adding NK cells to cultures with TuMTs and the triple culture with both MT types (**Figure 3A** and **Table S1**). In those cultures, the average growth of TuMTs was significantly lower than that of TuMTs in mono-cultures and TuMT-CarMT co-cultures (**Figure S4A**). Several TuMTs, especially in the triple cultures, shrank between day 2 and day 3 of the experiment. These shrinking MTs shared a few commonalities: (i) higher NK cell accumulation within the MT compartment and the TuMT itself, (ii) lower viability as shown by higher caspase 3/7 activity through live-cell fluorescence imaging (**Figure 3B**, left panel), and low intracellular ATP content, measured at day 3 of the experiment (**Figure S5**). TuMTs that grew in diameter had none or only a few NK cells on their surface or in the peripheral zone (**Figure 3B**, right panel). This heterogeneous tumor growth suppression can be attributed to (i) different levels of interaction between NK cells and TuMTs during the initial priming period and/or the first day (**Figure S6**), (ii) poor tumor invasion by NK cells, and/or (iii) immune escape of TuMTs (46).

Proteolytic shedding of MICA’s ectodomain is one of the major mechanisms used by tumor cells to escape from NK cell-mediated killing (44). The released sMICA has been shown to impair tumor cell recognition and cytotoxic activity of NK cells by direct blockage or by sMICA-induced internalization and degradation of NKG2D receptors (45). After confirming the membrane-bound expression of MICA on TuMTs (**Figure 2A**, left panel), we also measured the sMICA concentration released into the cell culture supernatant for different culture conditions. In agreement with the IF staining results for membrane-bound MICA/B (**Figure 2A**, right panel), we did not detect any sMICA in the mono-cultures of CarMTs or NK cells, as well as in the CarMT-NK cell co-cultures. In contrast, less than 5 pg/mL of sMICA were detected in mono-cultures of TuMTs in a 3-day experiment, which indicates the presence of MICA shedding (**Figure 3C**). Interestingly, MICA shedding was enhanced significantly in TuMT/NK cell co-cultures and in triple cultures, especially at day 3 of the experiment.

#### Tumor-Infiltrating NK Cells

**Figure 4:**
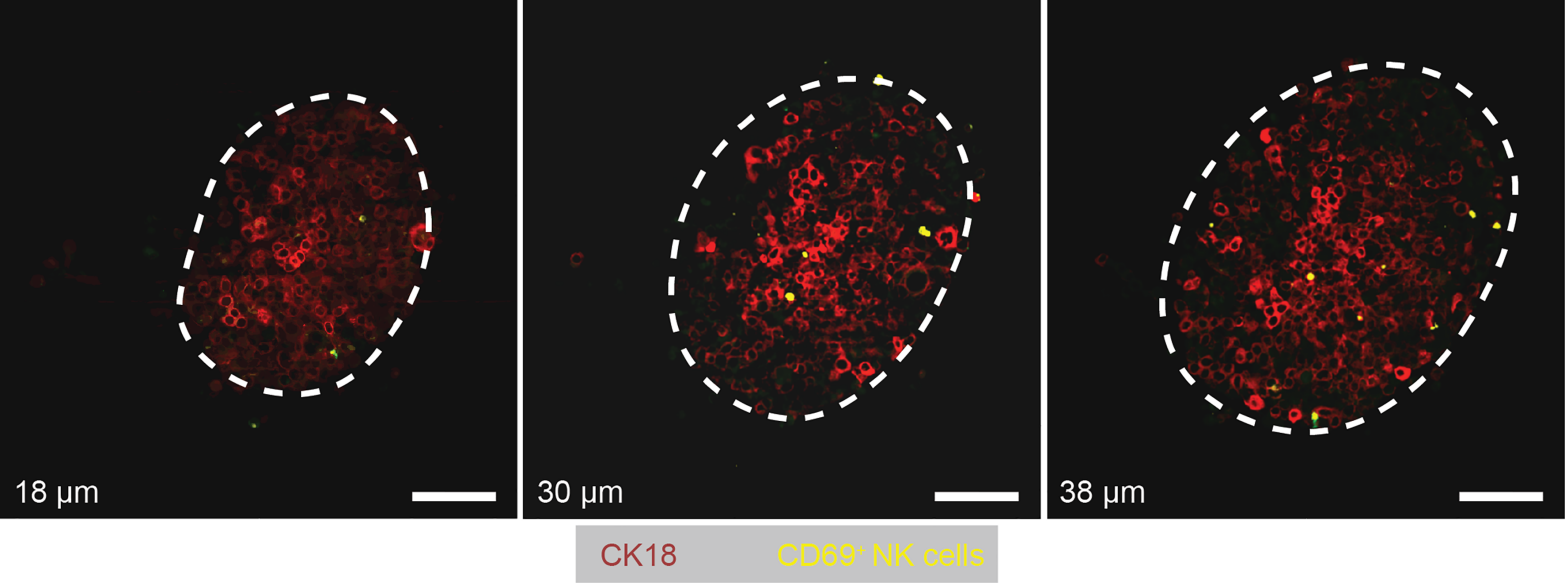
Images showing tumor-infiltrating NK cells at different Z-positions in a TuMT. The Z-depth – in reference to the bottom of the TuMT – is indicated at the bottom left of each image. White dashed lines indicate the outer border of the TuMT in the corresponding Z-plane. Scale bars: 100 μm.

As an additional endpoint analysis of the experiment, we fixed the MTs directly on-chip and stained them with CD69 and CK18 antibodies. CD69 is an activation marker for NK cells, while CK18 is an epithelium-specific cytoskeletal protein. CK18 plays a role in maintaining tissue integrity and was shown to be overexpressed in colorectal cancer tissues and cell lines, including the HCT116 cell line used in our work (47). It is important to note that only cells that were double positive for Green fluorescence and CD69 staining were qualified as CD69^+^ NK cells, as NK cells were stained with Cytopainter staining reagent prior to being seeded into the iMPS. We searched for tumor-infiltrating NK cells by taking Z-stacks of a total thickness of 190 – 200 µm and a Z-stack size of 2 μm using a confocal microscope. As shown in **Figure 4**, we found only a few NK cells that infiltrated the TuMTs across all examined MTs. Most of these tumor-infiltrating NK cells were CD69^+^ and resided within the few outermost cell layers of the TuMT.

#### Chemokine/Cytokine Signaling

**Figure 5:**
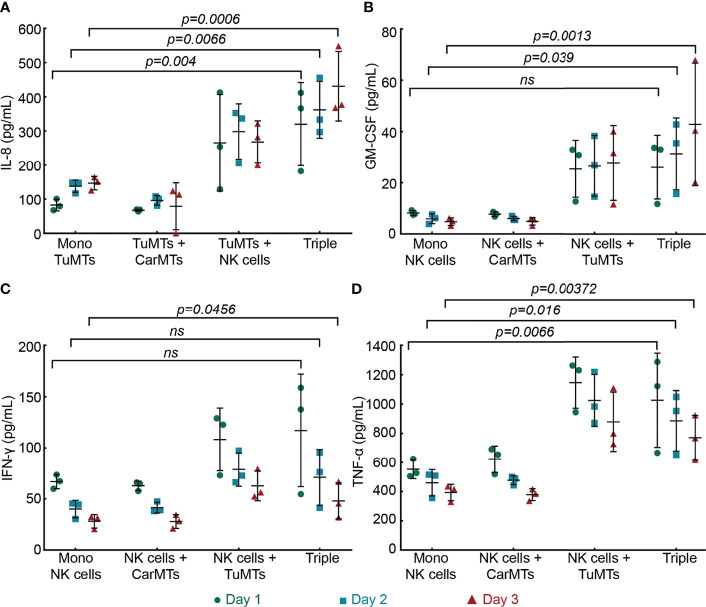
Quantification of the chemokines/cytokines **(A)** IL-8, **(B)** GM-CSF, **(C)** IFN-γ, and **(D)** TNF-α in the supernatant of different cell culture conditions over a 3-day experimental period (n = 3) (Mono, mono-culture; Triple, triple culture) (ns: not significant). Detailed statistical comparisons between conditions are shown in **Figure S8**.

We next investigated the chemokine/cytokine signaling in different culture conditions inside our MPS. To evaluate the response of TuMTs to NK cell exposure, we measured IL-8 in the cell culture supernatant in all cell culture conditions. IL-8 level has been proven to increase in many types of solid tumors, including colorectal tumor. An increased serum IL-8 content is currently considered a potential predictive marker of higher grade tumor burden and resistance to chemo- and immune-therapies (48). As shown in **Figure 5A**, mono-cultures of TuMTs produced increasing amounts of IL-8, ranging from 83 ± 17 pg/mL at day 1 to 138 ± 18 pg/mL at day 2, and 147 ± 19 pg/mL at day 3. In contrast to the levels measured for TuMT mono-cultures, IL-8 levels significantly spiked in co-cultures of TuMTs and NK cells. They slightly fluctuated in the TuMT-NK-cell co-cultures but increased steadily in triple cultures – from 320 ± 120 pg/mL at day 1 to 430 ± 100 pg/mL at day 3 – and remained significantly different from those observed in TuMT mono-cultures. Mono-cultures of NK cells and CarMTs consistently produced less than 10 pg/mL of IL-8 (**Figure S7A**).

As an indicator for indirect anti-tumor activity of NK cells, we measured the amount of GM-CSF, IFN-γ, and TNF-α, which were released by NK cells into the cell-culture medium. In the absence of NK cells, all these cytokines of interest were undetectable (**Figures S7B-D**). However, NK cells in mono-culture abundantly produced all three cytokines. The absence of other T cell-associated cytokines, e.g., IL-6, and IL-17 (data not shown), confirmed that NK cells were the only source of these cytokines in our system (**Figures 5B-D**). All cytokine levels dropped slightly over time in mono-cultures of NK cells, which is commonly observed when IL-15 was withdrawn from the cell culture medium (49, 50). The production of these cytokines was more extensive in co-cultures of TuMTs with NK cells, compared to mono-cultures of NK cells. However, all cytokines displayed different time-dependent dynamics. Over the experimental period, GM-CSF levels increased slightly in TuMT-NK cell co-cultures and triple cultures. In contrast, IFN-γ and TNF-α levels decreased slightly over time in all culture conditions. Interestingly, the IFN-γ level peaked at day 1 and dropped to a basal level within less than 2 days in the triple cultures, while there was no clear trend in TuMT-NK-cell co-cultures with respect to the basal level. TNF-α levels of all culture conditions that included TuMTs remained higher than of those without tumors until the end of the experiment (**Figure S8**).

Besides NK cells that (i) moved inside the iMPS with the flow (**Video S2**) and (ii) interacted with TuMTs (**Figures 3**, **4**), a portion of NK cells did accumulate inside cell enrichment zones during the experiment. This circumstance offered us the possibility to parallelly investigate the indirect tumor growth suppression of NK cells through soluble mediators, i.e., chemokines/cytokines. Therefore, in a different set of triple cultures, we removed all NK cells inside the cell enrichment zones on day 1. **Figure S9** shows the drop of GM-CSF, IFN-γ, and TNF-α levels after NK cell removal, while IL-8 levels increased over the next two days, as all TuMTs continued to grow, albeit slowly (**Figure S9A**). This experiment further confirmed the dependency of the system on NK-cell-mediated signaling.

#### NK-Cell-Induced Anti-Tumor Activity Effects on CarMTs

**Figure 6:**
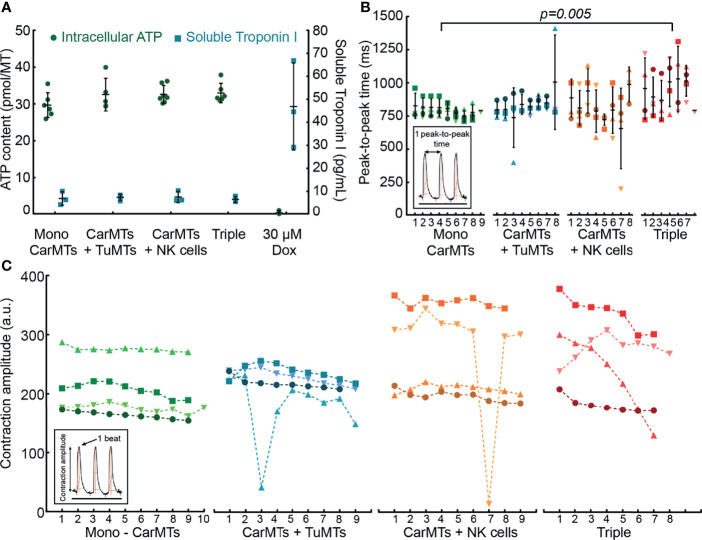
**(A)** ATP-contents of CarMTs (n = 6) indicating viability and average soluble Troponin I, produced by individual CarMTs under different culture conditions (n = 3) (Mono, mono-culture; Triple, triple culture; Dox: Doxorubicin hydrochloride). **(B)** Changes in beating patterns of CarMTs under different conditions, represented as peak-to-peak time between contractions (ms). The figure shows exemplary patterns of four CarMTs per culture condition. The numbering on the X-axis indicates the peak-to-peak interval count of individual CarMTs within a 20-seconds recording window (peak-to-peak intervals are shown in the insert graph.). **(C)** Contraction amplitudes of CarMTs under different culture conditions. The figure shows exemplary patterns of four CarMTs per culture condition. The numbering on the X-axis indicates the beat count of individual CarMTs within a 20-second recording window (beat counting is illustrated in the insert graph). For each culture condition, contraction amplitudes of the same CarMT were connected by a dashed line in chronological order. The same color code was applied for the same CarMT in both **(B, C)**.

Finally, we investigated the behavior of CarMTs for all described culture conditions by analyzing their physical interaction with NK cells, ATP-dependent viability, soluble Troponin I secretion, and beating patterns. The Troponin I level in patient serum is a clinically used biomarker that indicates cardiac injuries at elevated levels. Hence, we used soluble Troponin I as an indicator for health status of CarMTs in our iMPS. As shown in **Figure 6A** and **Figure S10A**, NK cells infiltrated CarMTs but did not negatively affect the viability of CarMTs under all culture conditions. Additionally, while CarMTs disintegrated after being exposed to 30 μM Dox for 3 days in a well-plate-based test, CarMTs in co-culture with NK cells and in triple culture on-chip remained intact (**Figure S10B**). The average Troponin I level per CarMT was lower than 10 pg/mL under all conditions in our iMPS as compared to the value obtained for Dox-treated MTs (47 ± 19 pg/mL per CarMT), which indicated that there was no structural damage of cardiac myocytes in the CarMTs (**Figure 6A**). Looking at the contraction profiles of the MTs, only a slight arrhythmia was observed in the CarMTs of CarMT-NK-cell co-culture (**Figure S11C**), while the CarMTs of the triple culture exhibited an obviously decreased beating rate (**Figure S11D**). In-depth analyses of the beating patterns of four exemplary CarMTs per culture condition revealed an increased average peak-to-peak time only in the CarMTs of the triple culture (**Figure 6B**). The majority of scrutinized CarMTs in the triple culture showed irregular contraction amplitudes as shown in **Figure 6C**. In fact, under this culture condition, CarMTs experienced highly elevated levels of both tumor-derived and NK cell-derived pro-inflammatory chemokines and cytokines, most importantly IL-8 and TNF-α (**Figures 5A, D**), that have been shown to negatively affect cardiac contractility *in vivo* (51, 52). Meanwhile, in CarMT-TuMT and CarMT-NK cell co-cultures, only one in four of CarMTs exhibited irregular contraction amplitudes, suggesting that detrimental effects on CarMTs may already be inflicted at a lesser extent by TuMTs or activated NK cells, or in other words, by lower levels of TuMT-derived IL-8 (**Figure 5A**) or NK cell-derived TNF-α (**Figure 5D**).

## Discussion

Despite the therapeutic potential of immune cell-employed ACT, there is still a large gap between *in-vitro* performance and *in-vivo* efficacy. This discrepancy mainly is due to a limited access to physiologically relevant tumor models and a lack of suitable *in-vitro* platforms for studying interactions between tumor models and immune cells. Interdisciplinary approaches will help to overcome these problems and increase the relevance of *in-vitro* screenings. While 3D tumor models offer more biological relevance (14, 53–56), iMPSs can provide physiological niches and critical cues for tumor models and immune cells to recapitulate physiological interaction (22, 24, 57–59). Although many initiatives show promising results, standardized iMPSs are still missing. Reasons may include the limited scalability of many academic approaches, the use of non-standardized and highly specialized tissue models, differences in screening protocols among laboratories, and the difficulty to transfer existing approaches to a broader community and clinical or industrial settings.

In this work, we developed an iMPS to study direct and indirect effects of anti-tumor NK cells on TuMTs and CarMTs. The inclusion of CarMTs into our iMPS allowed for a simultaneous assessment of potential off-target effects caused by anti-tumor NK cells. Interestingly, while a complete eradication of 3D TuMTs by NK cells was achieved in our static experiment, we observed heterogeneous tumor-killing activities by NK cells in our iMPS. This discrepancy shows how static culture conditions – where all cell components are forced to interact – can lead to an overestimation of ACT efficacy. Direct killing of TuMTs by NK cells was observed in our iMPS by a combination of different features: accumulation of NK cells in direct proximity of the MT, an increase in caspase 3/7 activity in tumor cells, and TuMT growth arrest or shrinkage. We also observed TuMTs that displayed a non-responsive phenotype within the same microfluidic channel. In such non-responsive TuMTs, growth and viability were not affected by the presence of NK cells (**Figure 3** and **Figure S6A**). As shown in **Figure S6B**, growth trajectories of TuMTs were determined by the level of direct interaction between TuMTs and NK cells within the first day of co-culturing rather than the number of NK cells in proximity of the TuMTs during the initial priming period. TuMTs that harbored large numbers of NK cells at day 1 grew slower or were subjected to growth suppression. Meanwhile, TuMTs harboring only a few NK cells at day 1 experienced less growth suppression that was mainly a consequence of the presence of NK cell-derived cytokines. The increased level of sMICA shedded from TuMTs (**Figure 3C**) may contribute to the observed ineffective NK cell-mediated tumor killing activity and heterogeneous tumor growth suppression.

Additionally, as shown in **Figure S6**, once the diameter of a given TuMT surpassed 500 μm, it was more likely to resist NK cell-induced growth suppression. It has been shown in other studies that TuMTs that are larger than 500 μm in diameter typically develop a hypoxic core (60, 61). Hypoxia induces hypoxia-driven adaptive mechanisms that promote tumor heterogeneity and survival while it imposes an immunosuppressive microenvironment on immune cells (62). Although the specific effect of hypoxia on NK cells remains elusive, it was shown to cause NK-cell dysfunction and to impair direct tumor-killing by tumor-infiltrating NK cells (63).

The chemokine/cytokine profiles of the on-chip cultures confirmed the reciprocal signaling between TuMTs and NK cells, indicating their interaction. We observed with all TuMTs that only a few NK cells infiltrated the TuMTs. Similarly, only low numbers of tumor-infiltrating NK cells were reported in different studies (46, 64, 65). Using whole-tissue sections of 112 patients and performing an *in-situ* quantification of immune cells, Halama *et al*. showed that NK cells were scarce in colorectal cancer tissue, even at early stages of the tumor development. NK cell invasion and retention in tumor tissue was low despite a high local level of chemokines, such as IL-8, and increased levels of IFN-γ and TNF-α in comparison to the mucosa adjacent to the tumor tissue (64). In another study, Rios-Doria et al. (66) developed xenograft models from different human tumor cell lines in humanized mice and quantified the presence of different immune-cell types within the tumor. Their results showed high infiltration levels for B-cells and dendritic cells, while tumor-infiltrating NK cells only amounted to between 1% and 5% of total tumor-infiltrating lymphocytes. Interestingly, the low number of NK cells – comparable to the number of tumor-infiltrating NK cells – was shown to induce resistance against NK cell-mediated killing in melanoma-resection-derived melanoma cell lines (67). To reveal the reasons for the resistance against NK-cell-mediated killing in our iMPS, extensive genomic and proteomic analyses will be required in future work.

We attributed the heterogeneous anti-tumor activity of NK cells to (i) different numbers and/or activation states of NK cells that could establish physical interactions with TuMTs within the first day of the experiment, (ii) chances of mutations within TuMTs that lead to immune-editing and eventually escape from NK cell-induced cell apoptosis (68), (iii) the development of tolerance for tumor cells by NK cells (69), or (iv) the activity suppression of NK cells by hypoxia and soluble factors shed from tumor cells (63, 70).

By including gravity-driven flow, our iMPS readily supported indirect, soluble-factor-mediated interaction between all included tissue models. This feature allowed us to simultaneously examine the response of TuMTs to NK cell-mediated killing activity and its impact on healthy CarMTs. A constant exposure of CarMTs to chemokines/cytokines, released by TuMTs-NK cells interaction – as shown in our iMPS – is difficult to realize with medium-conditioning approaches due to the short half-live times of IL-8 and TNF-α (half-live time of IL-8: 24 minutes, half-live time of TNF-α – 18.2 minutes) (71).

Interestingly, we did not detect any structural damages of cardiac myocytes in CarMTs for all our on-chip culture conditions. Nevertheless, the high level of chemokine and cytokine release by both TuMTs and NK cells upon interaction in the triple culture significantly reduced the beating frequency and altered the contraction amplitude of CarMTs. This observation is in agreement with *in-vitro* and *in-vivo* investigations by Buoncervello et al. (52). In their *in-vitro* analysis, the authors dosed cardiac myocyte cultures with different inflammatory chemokines/cytokines, including IL-8, IFN-γ, and TNF-α for 48 hours. They reported an absence of cell death but various “severe phenotypic changes” in chemokine/cytokine-treated cardiac myocytes, indicating a dysfunction of contractile cytoskeletal elements. They also provided evidence on the link between colorectal tumor-induced heart systolic dysfunction and chronic systemic inflammation in their follow-up *in-vivo* experiment (52). Similar to our results, they did not detect any elevation of Troponin I in animal plasma across all conditions. Not many studies have yet investigated the risks associated with NK cell-based ACT so that NK cells are generally considered to cause less side effects than T-cells (72). However, this consideration may be due to the fact that suitable tissue models and testing platforms that could reveal more subtle adverse effects are still lacking. Moreover, solid tumors can alter the immune response and other signaling pathways in ways that can lead to unexpected damages to other organs. Therefore, more systemic approaches and better tools are needed for researchers to address these open questions.

## Conclusion

In summary, we presented a simple and user-friendly iMPS that offers: (i) long-term triple culture of 3D TuMTs with anti-tumor NK cells and healthy CarMTs, (ii) microscopy-based observation of direct TuMT-NK cell interaction and evaluation of the spontaneous beating activity of CarMTs, (iii) collection of the cell-culture supernatant for chemokine/cytokine profiling, and (iv) harvesting of all tissue models for endpoint analyses. This proof-of-concept work is aimed at demonstrating the potential and versatility of iMPSs for use in immuno-oncology research, especially for early *in-vitro* validation and safety assessment of therapy approaches. More in-depth investigations regarding the growth inhibition of TuMTs, the specific receptor-ligand interactions involved in NK cell-mediated tumor killing, and more extensive profiling of the signaling-molecule repertoire remain topics for future work.

## Data Availability Statement

The raw data supporting the conclusions of this article will be made available by the authors, without undue reservation.

## Author Contributions

ON and PM conceived the approach and designed the experiments. TS established the pipeline to obtain human UCB samples. WW processed the UCB samples and prepared CD34^-^ fractions of UCB. JL differentiated cardiac myocytes from human iPSCs and developed protocols for CarMT formation. ON performed all other experiments and analyzed the data. ON, PM, CL, and AH wrote the paper. All authors contributed to the article and approved the submitted version.

## Funding

This work was financially supported by the two Cantons of Basel through the project (PMB-02-17) granted by ETH Zürich.

## Conflict of Interest

The authors declare that the research was conducted in the absence of any commercial or financial relationships that could be construed as a potential conflict of interest.

## Publisher’s Note

All claims expressed in this article are solely those of the authors and do not necessarily represent those of their affiliated organizations, or those of the publisher, the editors and the reviewers. Any product that may be evaluated in this article, or claim that may be made by its manufacturer, is not guaranteed or endorsed by the publisher.
