# Supplementary material for: An Immunocompetent Microphysiological System to Simultaneously Investigate Effects of Anti-Tumor Natural Killer Cells on Tumor and Cardiac Microtissues
Source: Front Immunol. 2021 Dec 2;12:781337. doi: 10.3389/fimmu.2021.781337 (PMC8675866; doi:10.3389/fimmu.2021.781337)
Supplement: Supplementary file 1 [file DataSheet_1.pdf]

An immunocompetent microphysiological system to simultaneously investigate effects of anti-tumor natural killer cells on tumor and cardiac microtissues

Supplementary Material

Supplementary Table

Table S1. Average tumor MT growth under different culture conditions (Mean diameter ± SD in µm; *n* = 18 MTs)

Culture condition	Day 1	Day 2	Day 3
Mono-culture	83 ± 38	158 ± 52	193 ± 55
TuMTs + CarMTs	108 ± 13	163 ± 16	205 ± 20
TuMTs + NK cells	76 ± 34	114 ± 39	135 ± 41
Triple culture	96 ± 32	120 ± 37	148 ± 68

Supplementary Videos

Video S1. An overview of tissue model formation, chip loading and operation, and the biological concept of this study.

Video S2. NK cells (labeled in green) moved with the flow through a MT compartment (time = 35 seconds).

Supplementary Figures

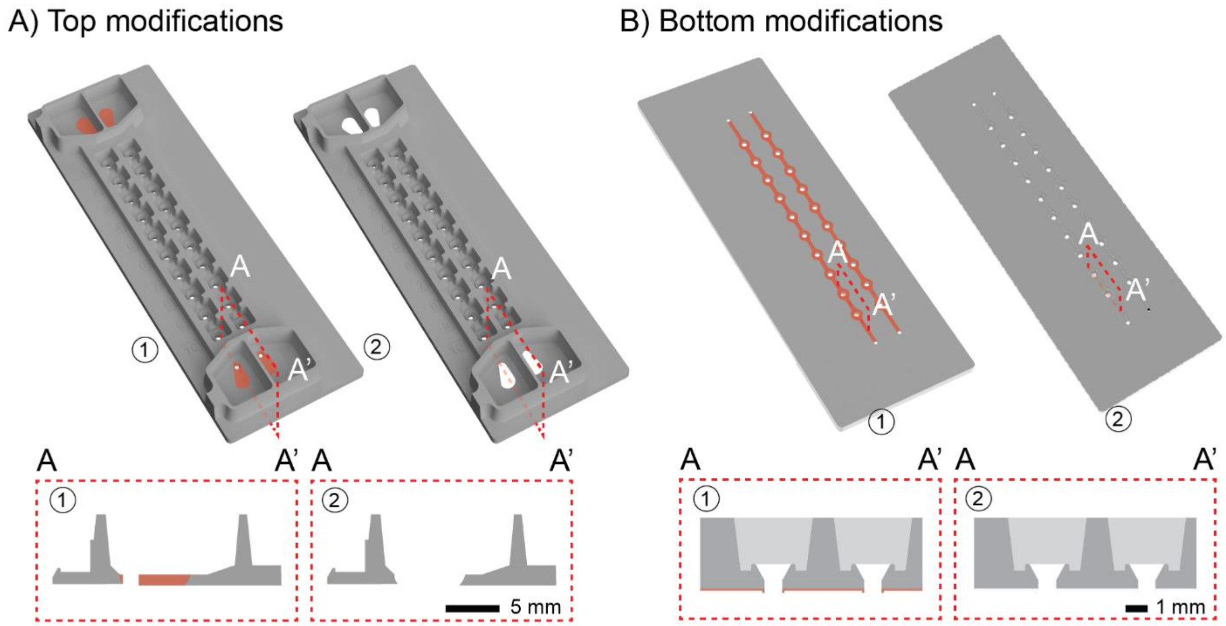

Figure S1. Detailed modifications of the original Akura™ Flow platform in this work: **A)** Introduction of the drop-shaped cell-enrichment zone in the original medium reservoirs and **B)** Removal of the barrier structures in the MT compartments. For each panel A and B, subfigure ① and ② show cross-sections of the areas of interest before and after the corresponding modifications, respectively. The areas labeled in red were removed by micro-milling.

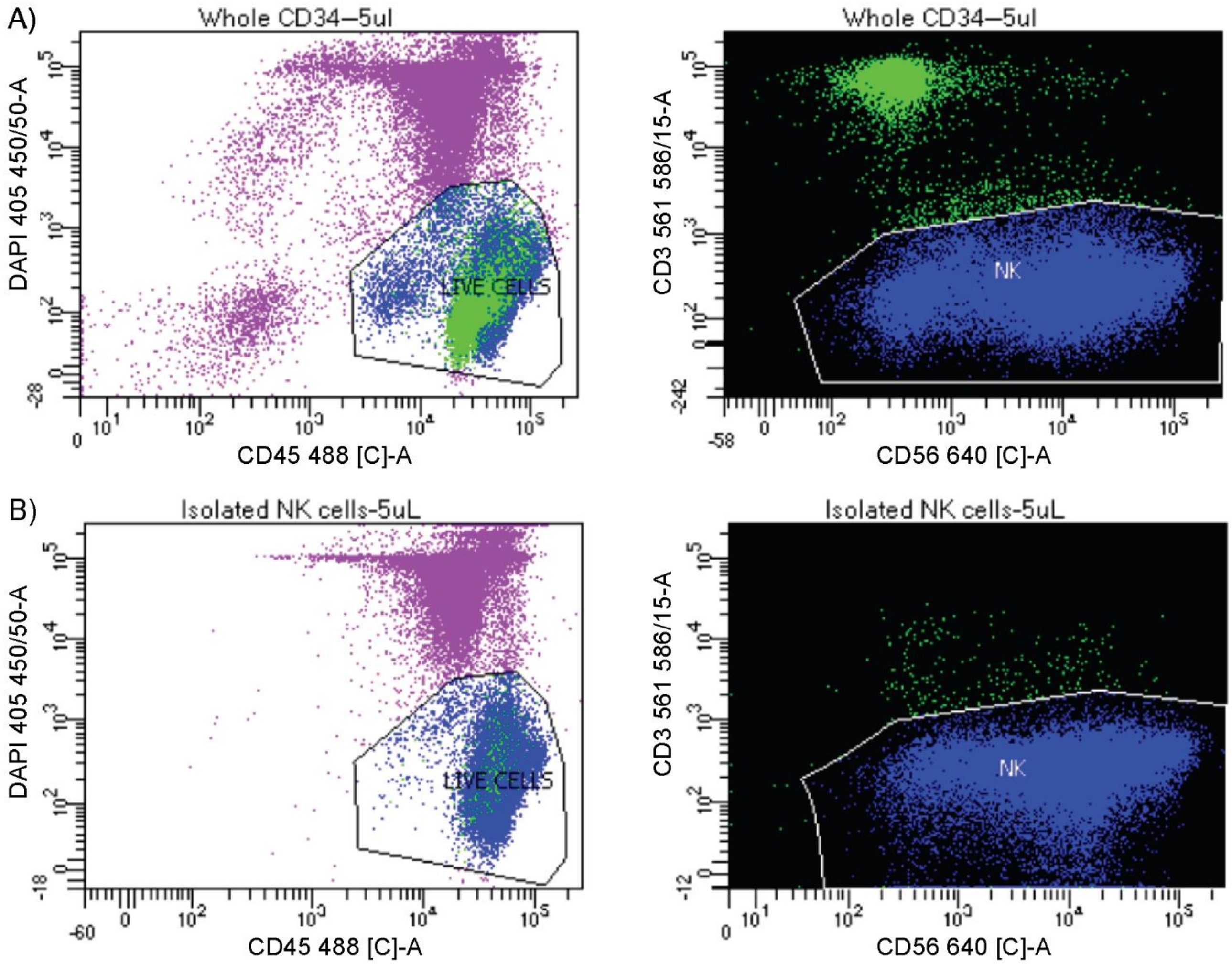

Figure S2. Flow cytometry analysis of **A)** $CD34^-$ fraction of UCB and **B)** isolated NK cells. Forward-scatter (FSC) versus side-scatter gating (SSC) was performed to remove cell debris and FSC-width (FSC-W) versus FSC-area (FSC-A) gating was performed to further remove doublets in further analyses (data not shown). After gating for live nucleated cells ($DAPI^-/CD45^+$), NK cells ($CD3^-/CD56^+$) were distinguished from T cells ($CD3^+/CD56^-$) using a CD3 versus CD56 two-parameter plot.

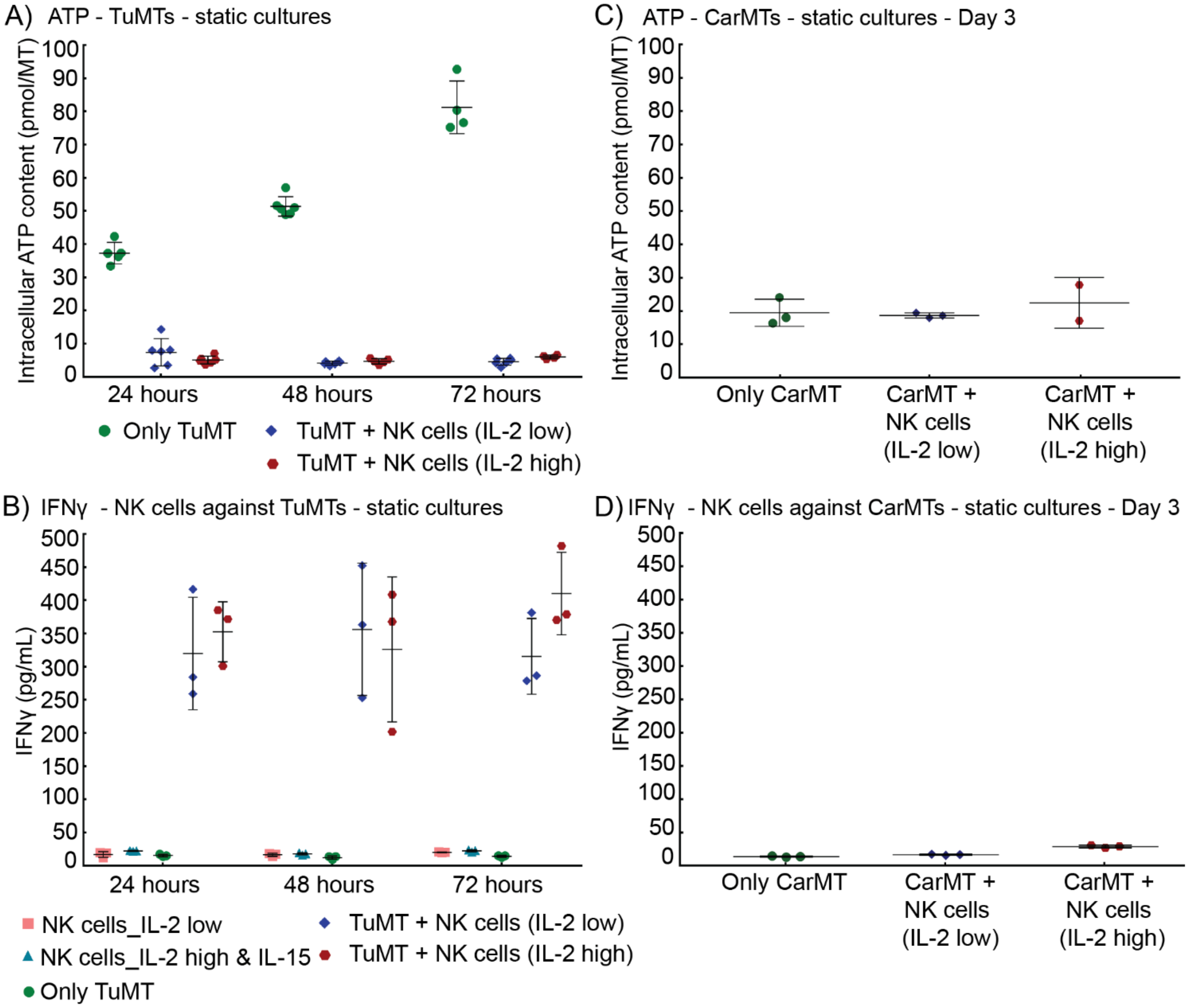

Figure S3. Results of characterization experiments on a well plate, under static conditions, showing specific killing activity of NK cells against TuMTs. **A)** ATP measurements of TuMTs (n = 6 MTs) and **B)** IFN-γ measurements in different culture conditions over 3 days (n = 3). **C)** ATP measurement of CarMTs (n = 3 MTs) and **D)** IFN-γ measurements in different culture conditions at day 3 of the experiment (n = 3). NK cells used under low-IL-2 conditions were maintained in NK cell growth medium – supplemented with 200 U/mL IL-2 – before use. Meanwhile, NK cells used under high-IL-2 conditions were cultured for 5 days in NK-cell-activating medium that contained 1000 U/mL IL-2 and 20 ng/mL IL-15 before the experiment.

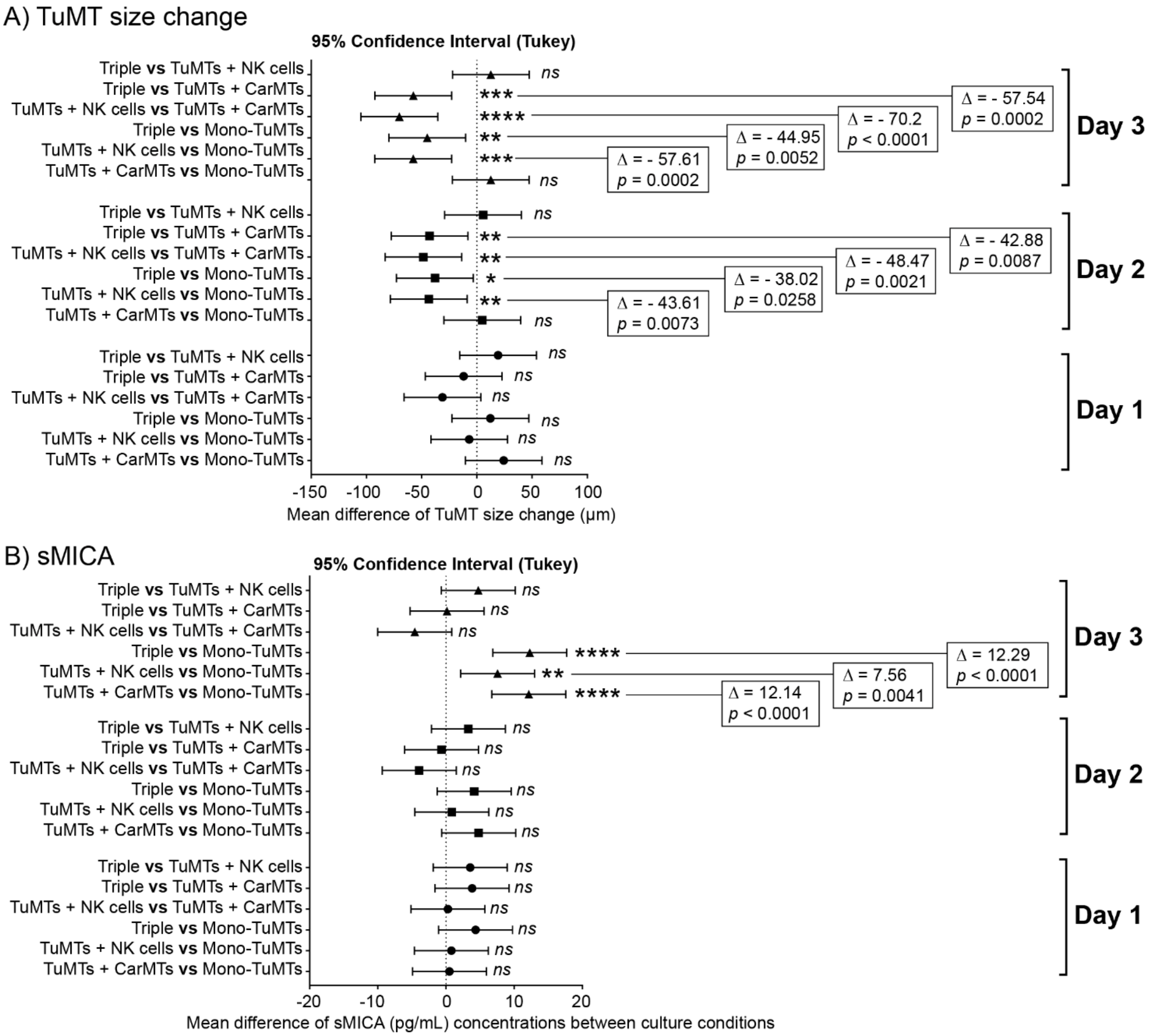

Figure S4. 95% confidence intervals (CIs) for the difference between the mean values of **A)** TuMT size changes and **B)** sMICA concentrations for different culture conditions (Mono: mono-culture, Δ: Mean difference).

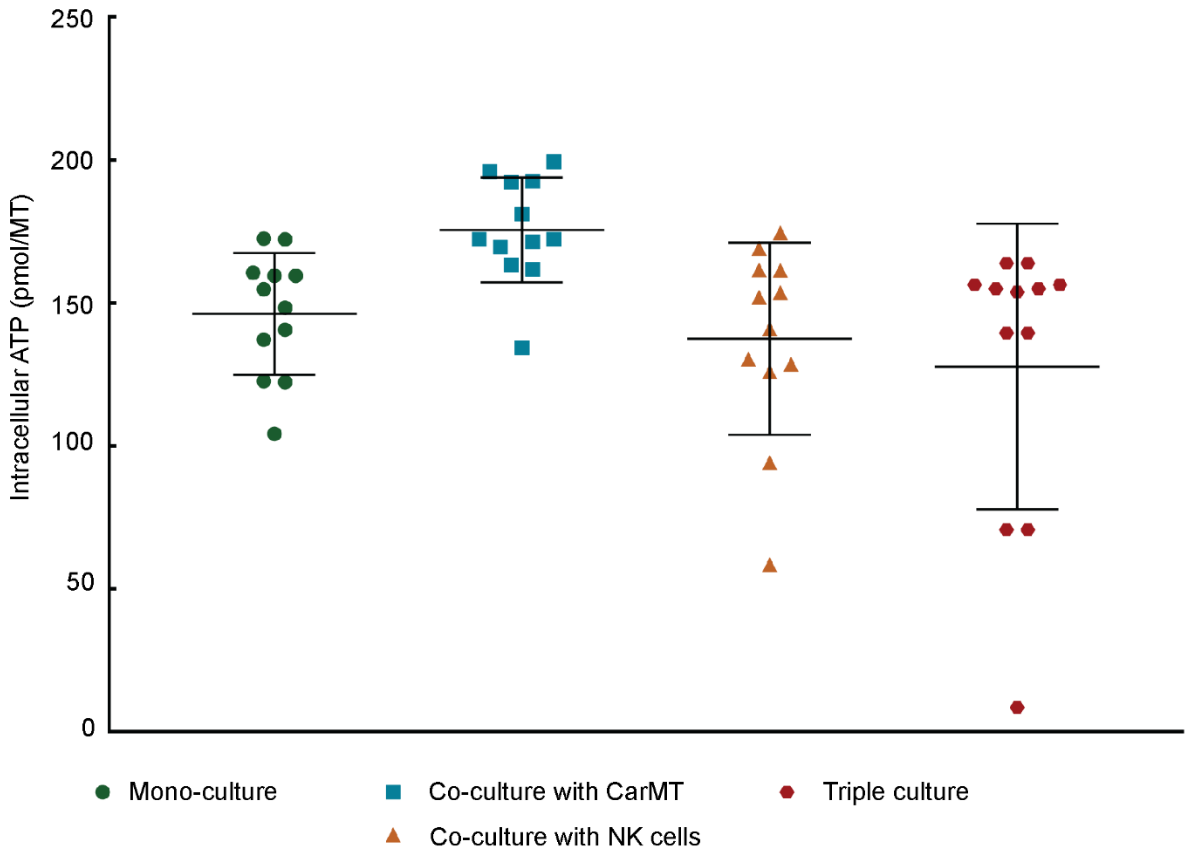

Figure S5. ATP measurements of TuMTs under different culture conditions (n = 12 MTs) after 3 days of on-chip culturing.

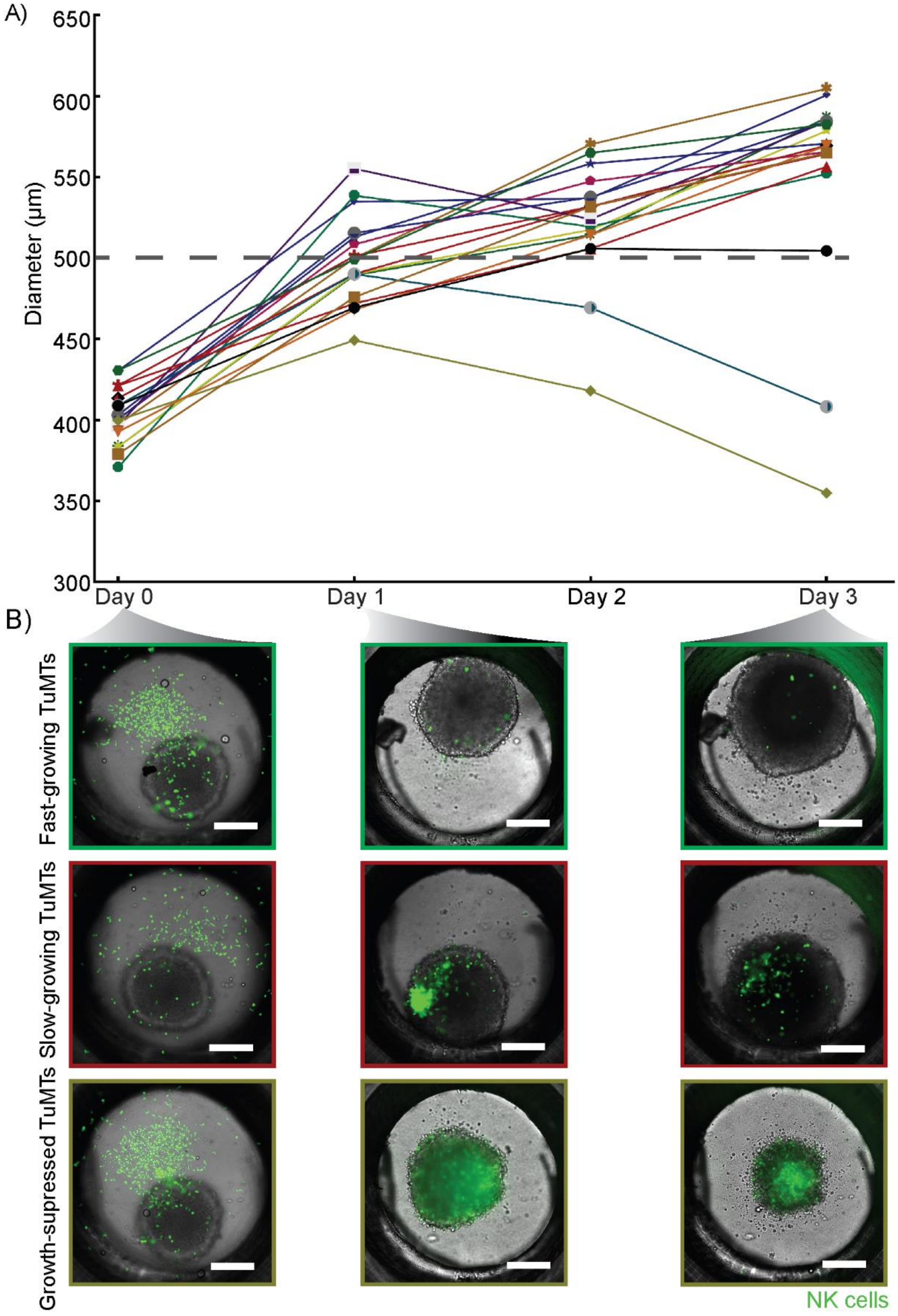

Figure S6. Heterogeneous growth of TuMTs in on-chip triple cultures. **A)** Growth curves of 18 TuMTs indicates heterogeneous growth patterns/growth suppression observed from day 1 of the experiment. **B)** Fluorescence images of exemplary TuMTs the growth of which was differently affected by cytokine-activated NK cells (1000 U/mL IL-2 and 20 ng/mL IL-15). Scale bars: 200 µm.

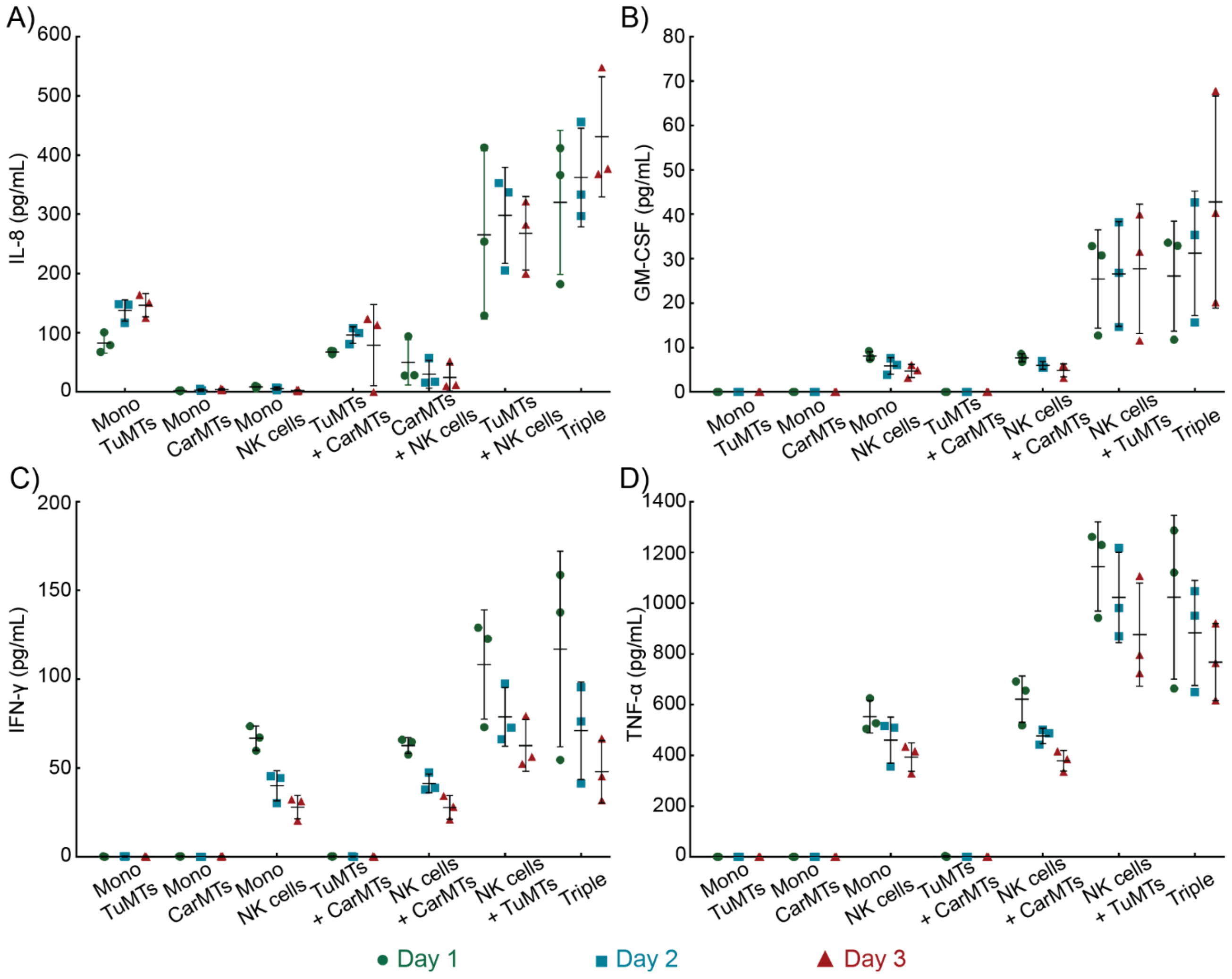

Figure S7. Cytokine profiles for all on-chip culture conditions in the supernatant over a 3-day experimental period (n = 3): **A)** IL-8, **B)** GM-CSF, **C)** IFN-γ, and **D)** TNF-α (Mono: mono-culture, Triple: triple culture).

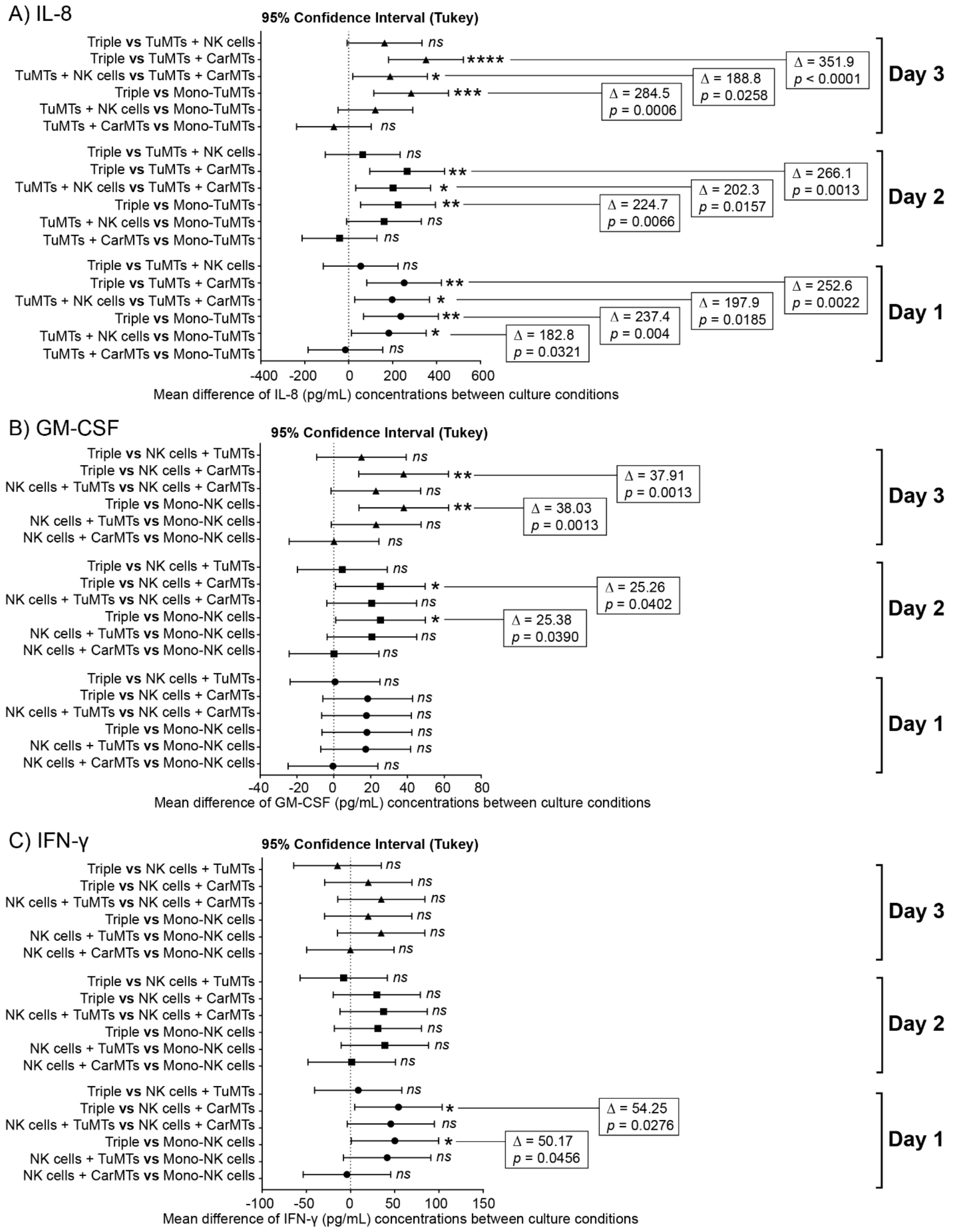

Figure S8. Statistical difference of **A)** IL-8, **B)** GM-CSF, and **C)** IFN-γ concentrations for different culture conditions as shown by 95% confidence intervals (CIs) of the difference of the mean values (Mono: mono-culture, Δ: Mean difference).

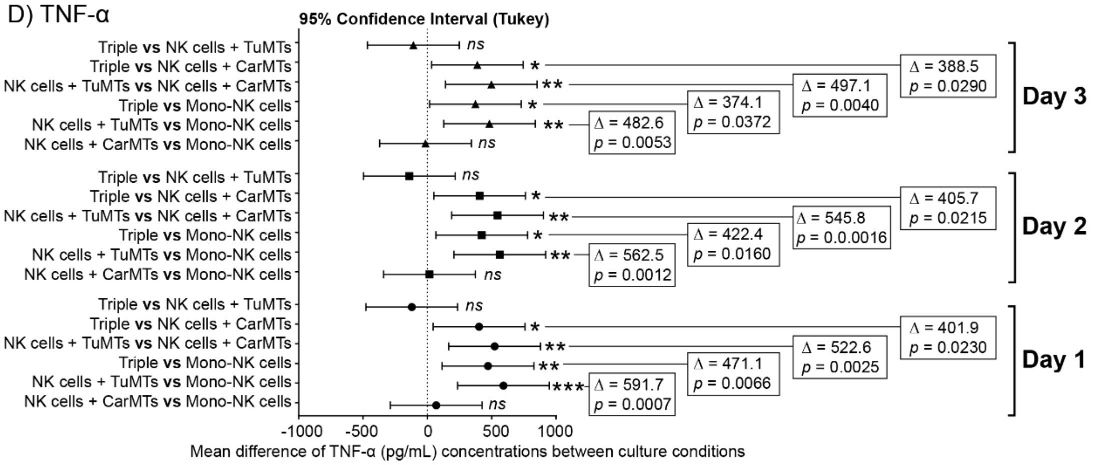

Figure S8 (cont.). Statistical difference of **D)** TNF-α concentrations for different culture conditions as shown by 95% confidence intervals (CIs) of the difference of the mean values (Mono: mono-culture, Δ: Mean difference).

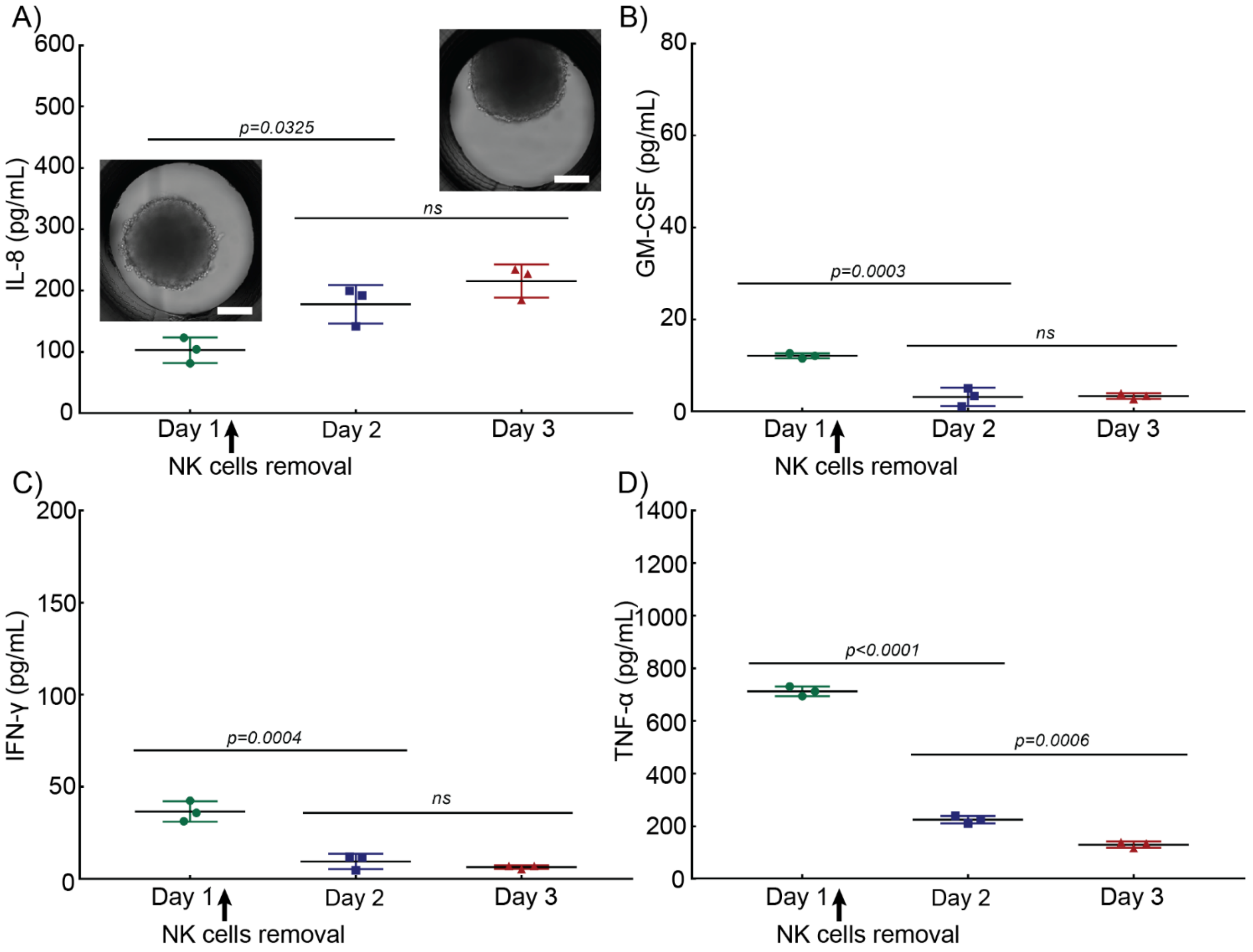

Figure S9. Cytokine levels in the supernatants of triple cultures after removal of NK cells (n = 3) at day 1: **A)** IL-8. IL-8 level slightly increased after NK-cell removal as TuMTs continued to grow (inset images); **B)** GM-CSF; **C)** IFN-γ; and **D)** TNF-α.

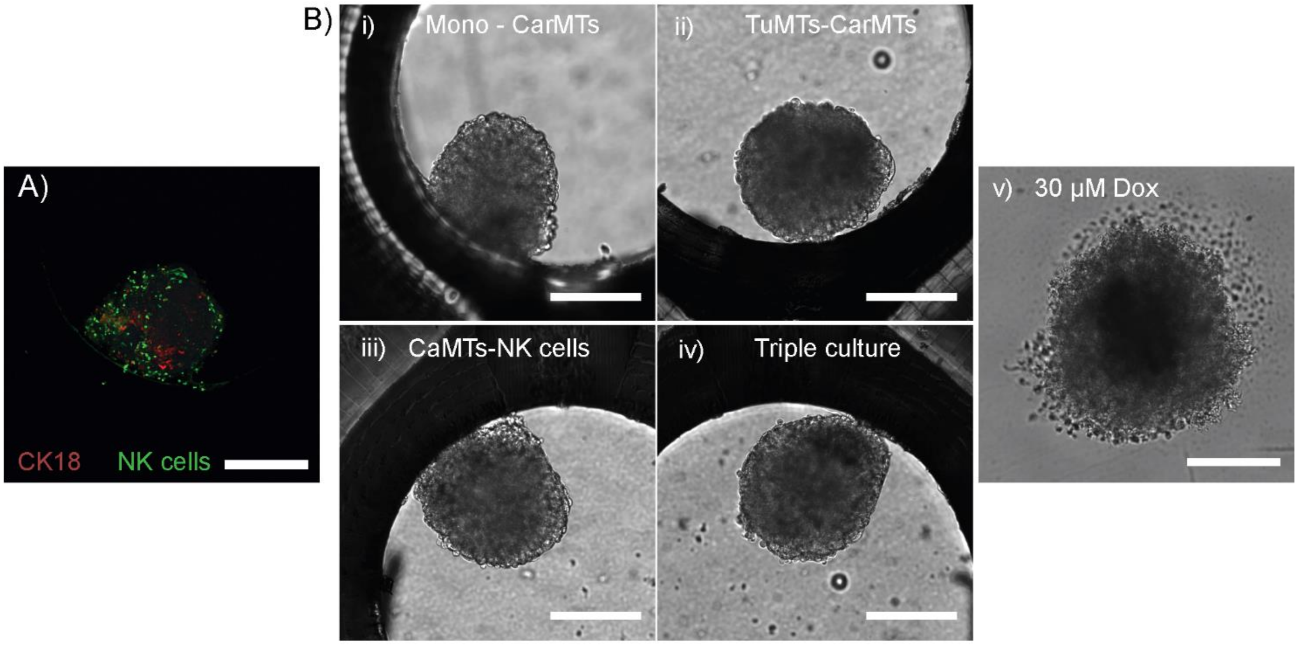

Figure S10. A) NK cells accumulated at the surface and within CarMT. Scale bar: 200 μm. CK18 staining indicated the presence of a small number of immature cells in iPSC-derived CarMTs. **B)** Bright-field images of representative CarMTs under different culture conditions (Mono: Mono-culture): (i-iv) show the morphological difference to Dox-treated CarMTs (v) (Dox: Doxorubicin hydrochloride). Scale bars: 200 μm.

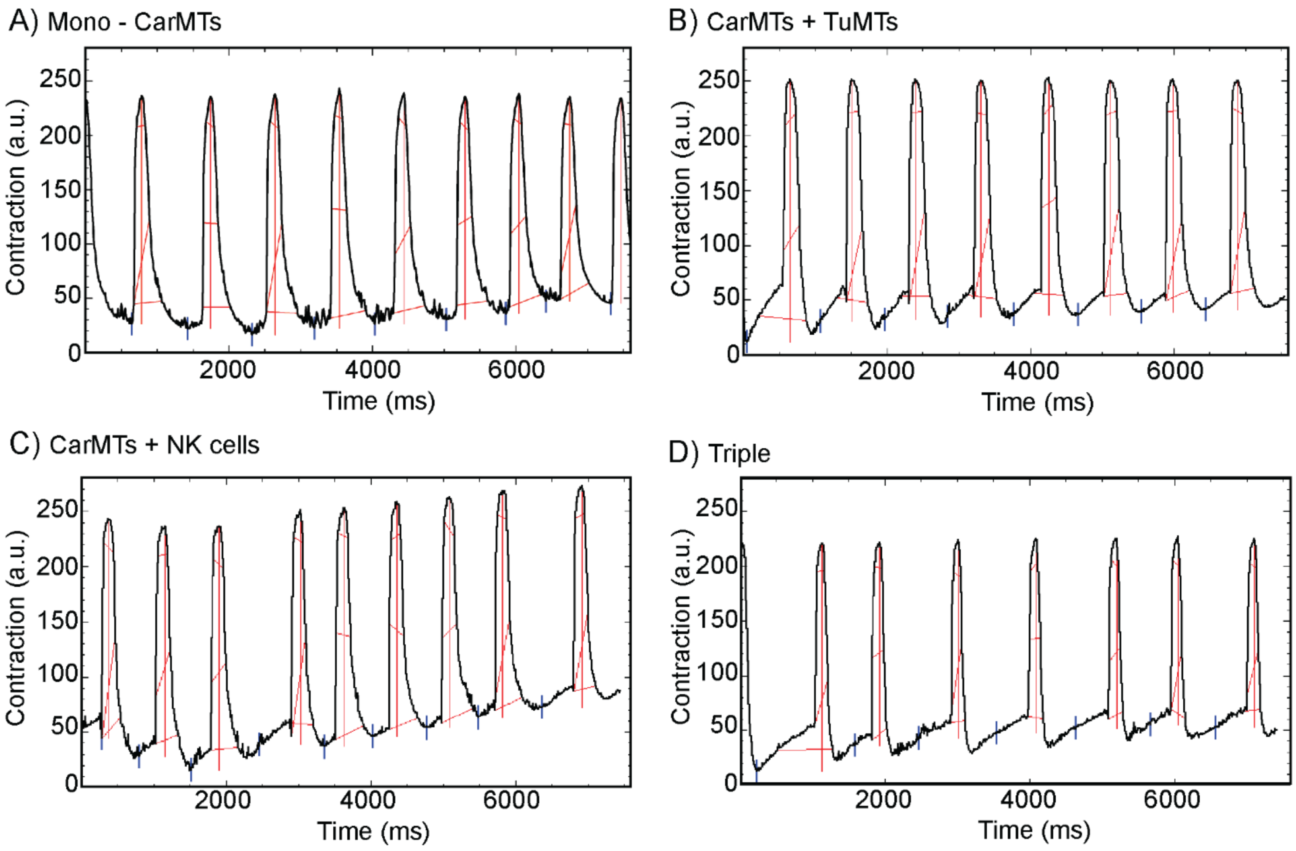

Figure S11. Contraction patterns of CarMTs under different culture conditions: **A)** Exemplary CarMT in mono-culture of only CarMTs, **B)** Exemplary CarMT in TuMTs-CarMTs co-culture, **C)** Exemplary CarMT in CarMTs-NK cells co-culture, and **D)** Exemplary CarMT in triple culture. (Mono: mono-culture, Triple: triple cultures).
